# Epilepsy and Its Treatment

**Published:** 1906-12-22

**Authors:** W. Aldren Turner

**Affiliations:** Physician to Out-patients, King's College Hospital, and National Hospital for Epilepsy and Paralysis, Queen Square


### EPILEPSY AND ITS TREATMENT.

### BEING AN ABSTRACT OF A LECTURE

By W. ALDREN TURNER, M.D.(Edin.), F.R.C.P.(Lond.), Physician to Out-patients, King's College Hospital, and National Hospital for Epilepsy and Paralysis, Queen Square.

THERE is no single specific remedy in the treatment of epilepsy, although the alkaline salts of bromine come nearest to this definition.

The bromides may act on epileptic fits by arresting the seizures, temporarily or permanently, and within a short period of their administration. In favourable cases this result ensues within a short time—under one year in 50 per cent.—of the commencement of treatment.

Secondly, the bromides may induce a lessening in the severity and frequency of the seizures.

The statistics of this hospital show that about 25 per cent. of the cases fall under the first head and another 25 per cent. under the second. When the bromide salt is stopped, the fits usually recur.

There is a third class, in which the bromides exert no influence whatever upon the seizures. This contains the remaining 50 per cent. From that statement it is evident that the treatment of epilepsy by the bromide medication is not very satisfactory, but there is no other drug so useful, and therefore I desire to discuss how best to administer bromides in the treatment of epilepsy. Most physicians who have to treat this disease have their own methods of doing so. I think, in the first place, that the bromides are given in many cases in too large quantities. In France, and in America, and sometimes here, very large doses of the bromides are administered and a condition of acute bromism is produced. It is hardly a scientific way to treat disease by producing the toxic effects of the drugs used. Bromides ought not to be given in doses of more than 60 grains in twenty-four hours; 30 grains daily is a minimum dose, 60 grains the maximum, and 45 a medium dose. There are, however, certain indications which may guide you as to which dose is most satisfactory to give, and I would lay down the following general directions. If the case is recent and the fits not more frequent than once every few months or once or twice a year and there has been no previous treatment—a virgin case of epilepsy—it is best to begin with a 30-grain dose, and if no amelioration results, proceed to 45-grain doses and hold the 60 grains in reserve for special circumstances. My experience is that it is better to give one large dose at bedtime, especially if the fits occur during sleep—the so-called nocturnal type—the salt used being either the potassium or the sodium bromide. If the fits occur both by day and night—the major fit by night and the minor by day—it is better to prescribe 45 grains—30 grains being given at bedtime and 15 grains in the morning.

How soon should you expect the benefits of treatment to ensue? This is a difficult point to answer specifically. On looking through the cases which have been treated at this hospital, one finds that in those in which the treatment proved successful the fits were arrested within the first year of treatment.

Are there any features of epilepsy which indicate whether a case is going to be successfully treated? The following are favourable aspects in any given case:—

1. The commencement of the disease about puberty.
2. Late or senile epilepsy, that form of the disease which occurs after 45 years of age.
3. Cases with infrequent fits—*e.g.* one or two a year, or those in which a year or more intervenes between the fits.
4. The absence of marked mental degeneration or epileptic dementia.
5. Absence of well-marked stigmata of degeneration.

Most cases are of a much more severe type and present unfavourable signs, such as—

1. The commencement of the disease under five years of age.
2. Great frequency of seizures—*e.g.* one or two a day, two or three a week, three or four a month.
3. A combination of major and minor fits.
4. Marked mental deterioration. If you have these types of epilepsy to treat you may give from the outset a bad prognosis.

Should the bromides fail in arresting the seizures, do any other remedies exist which may take their place?

Borax was originally introduced by Sir William Gowers, and his experience of it is, on the whole, favourable. The experience of others who have used it is, I believe, satisfactory; but mine is less so. In doses of 10 grs. it may be given in combination with the bromides.

Belladonna is an old remedy in the treatment of epilepsy, and before the introduction of the bromides was constantly used in the treatment of this disease. It has now largely passed out of use, but every now and again satisfactory results will be obtained from doses of ten to fifteen minims of the tincture.

Opium is now only used in the opium-bromide method of Flechsig. During six weeks the patient is given increasing doses of opium until he is taking about ten or twelve grains of the extract of opium daily. At the end of this time the opium is suddenly stopped and large doses of bromide of potassium are substituted, the dose of which is gradually diminished during the course of the following three months. Some cases respond well to this treatment. Some have serious accidents as a result, and the cases in which I have used the method have shown no improvement.

Strychnine is a remedy which has done great service in the treatment of epilepsy, especially in the nocturnal type.

Intestinal antiseptics, as salicylate of bismuth and beta-naphthol, may be useful when administered either separately or in combination with bromides; but as few attacks of epilepsy are due to intestinal intoxication, the treatment of the disease on these lines is not likely to prove successful.

The bromides in combination with various other remedies may be of service. Bromide with nux vomica and a little arsenic is often useful. This tonic treatment seems just to give the necessary stimulus to the nervous system. In cases of epilepsy associated with mitral disease or irregular or intermittent action of the heart you will get satisfactory results from the addition of the tincture of digitalis.

The treatment of epilepsy by means of organic preparations, such as thyroid, cerebrine, thymus gland, etc., has been proved to be useless. Thyroid increases the tendency to convulsions, although it may temporarily diminish dementia.

The dietetic treatment is an aid to bromide medication. A non-stimulating diet is more efficacious that a diet containing meat. It is a mistake, however, to remove all forms of animal food from the diet of epileptics. The epileptic requires a certain amount of proteid matter, and the type of diet which suits the epileptic best is one containing a minimum of animal food with a maximum of vegetable. I find the purin-free diet the most useful in the early stages of the disease and as an adjuvant to the bromides. Circumstances which favour the use of this diet are: First, the major type of epilepsy with absence of the minor seizures; secondly, increase of the arterial tension as occurs in many cases of arterio-sclerosis. The dose of bromide may be reduced in combination with this diet, and that is an important factor in the treatment of many cases of epilepsy.

